# Supplementary material for: Mitochondrial DNA Copy Numbers and Lung Cancer: A Systematic Review and Meta-Analysis
Source: Int J Mol Sci. 2025 Jul 10;26(14):6610. doi: 10.3390/ijms26146610 (PMC12294451; doi:10.3390/ijms26146610)
Supplement: Supplementary file 1 [file ijms-26-06610-s001.zip › ijms-3728343-supplementary.pdf]

Supplementary Table S1. Search strategy

**Database**	Search details
**PubMed**	("dna, mitochondrial"[MeSH Terms] OR ("dna"[All Fields] AND "mitochondrial"[All Fields]) OR "mitochondrial dna"[All Fields] OR ("mitochondrial"[All Fields] AND "dna"[All Fields])) AND "copy"[All Fields] AND ("number"[All Fields] OR "numbers"[All Fields]) AND (("lung neoplasms"[MeSH Terms] OR ("lung"[All Fields] AND "neoplasms"[All Fields]) OR "lung neoplasms"[All Fields] OR ("lung"[All Fields] AND "cancer"[All Fields]) OR "lung cancer"[All Fields]) AND ("risk"[MeSH Terms] OR "risk"[All Fields]))
**Web of Science**	(ALL=(mitochondrial DNA copy number)) AND ALL=(lung cancer risk)
**Scopus**	(ALL (mitochondrial AND dna AND copy AND number) AND ALL (lung AND cancer AND risk))

Supplementary Table S2: New Castle Ottawa Scale of the studied selected of the Case-Control studies.

Case-Control Study	Adequate definition of cases	Representativeness of cases	Selection of controls	Definition of controls	Comparability		Ascertainment exposure	Same method of ascertainment for cases and controls	Non-Response rate	Total quality score
					important factor	additional factors				
**ncDNA mt**										
Kennedy GT, 2022 USA, (113)	0	1	1	1	1	1	1	1	1	8
Meng S, 2016 USA, (388)	1	1	1	1	1	1	1	1	1	9
Kim C, 2014, China, USA, Finland (463)	0	1	1	0	1	1	1	1	1	7
Hosgood HD III, 2010 Finland (562)	1	1	1	1	0	0	1	1	1	7
Bonner MR, 2009 China, (581)	1	1	0	0	1	1	1	1	0	6
